# The experience of foot problems and decisions to access foot care in patients with rheumatoid arthritis: a qualitative study

**DOI:** 10.1186/s13047-017-0188-3

**Published:** 2017-01-25

**Authors:** Oonagh Wilson, John Kirwan, Emma Dures, Enid Quest, Sarah Hewlett

**Affiliations:** 1 Faculty of Health and Applied Sciences, University of the West of England, Bristol, UK; 2 School of Clinical Sciences, University of Bristol, Bristol, UK; 3 Rheumatology Department, Bristol Royal Infirmary, Bristol, UK; 4 Academic Rheumatology Unit, The Courtyard, Bristol Royal Infirmary, Bristol, BS2 8HW UK

**Keywords:** Foot problems, Rheumatoid arthritis, Foot care, Patient experience, Qualitative methods

## Abstract

**Background:**

Although foot problems are common in rheumatoid arthritis (RA), the consequences of foot problems from the patient perspective have not been fully explored. The aims of this study were to explore the experience of foot problems and decisions to access foot care services or not in patients with RA.

**Methods:**

Semi structured, one-to-one interviews with patients recruited from 2 UK rheumatology units, purposively sampled for self-reported foot problems and a range of personal/disease characteristics. Inductive thematic analysis was used, with rigour provided by multiple independent analysers. Emerging themes were discussed and agreed by all authors.

**Results:**

Twelve patients participated: 7 female; mean age 56 years (29–72); mean disease duration 12 years (2–27), 5 had accessed foot care services. The ‘Impact’ of foot problems was substantial and formed the underpinning theme, comprising three organising themes: ‘Foot symptoms’; ‘Consequences’; and ‘Cost’. Foot symptoms such as pain and numbness required self-management, and affected daily life (walking, working) leading to social and emotional costs. The global theme, ‘Decision to access foot care or not’, also comprised three organising themes: ‘Access perceived unnecessary’ (no problem, can cope); ‘Access hindered by patients’ perception’; and ‘Access supported by patient and clinician’. Decisions to access foot care or not were complex and influenced by patient beliefs regarding possible treatments and how to access these, and hindered by patient perceptions that their feet were ignored by rheumatology clinicians. Positive experience of foot care encouraged continued utilisation but negative experiences contributed to patients’ decisions to discontinue foot care services.

**Conclusions:**

Foot problems are important issues for patients and impact on many aspects of their physical, social and emotional lives. Patients who had accessed foot care services prioritised their foot problems as an important health care need. However, for others who would like foot care services, personal knowledge and values, and perceived barriers in clinical practice, appear to interact to inhibit foot care access. The extent which these interactions affect overall access to foot care in RA patients in general now needs to be quantified to help to inform and improve the effectiveness of the organisation and delivery of foot care.

**Electronic supplementary material:**

The online version of this article (doi:10.1186/s13047-017-0188-3) contains supplementary material, which is available to authorized users.

## Background

Rheumatoid arthritis (RA) is a chronic autoimmune condition causing symmetrical inflammatory poly-arthritis, sometimes with additional systemic features. The initial clinical presentation of RA is most often symmetrical pain, swelling and stiffness of the joints, often including the feet [1]. Over 90% of patients with RA are reported to experience foot problems at some time during their disease trajectory [2, 3] probably a result of a combination of the disease process and altered foot mechanics [4]. The additional mechanical stresses of walking in a weakened musculoskeletal environment can lead to pain, deformity, deterioration in walking distance, reduced activity levels and general wellbeing [3, 5–7]. Development of secondary skin lesions and extra-articular features add to the complexity of foot problems in RA [8–10]. These raise issues for patients regarding their ability to obtain accommodative footwear that is aesthetically acceptable to them [11, 12]. Functional impairments can also restrict patients’ ability to participate in foot health self-care [13].

National guidelines for managing RA [14] call for an annual review of the feet yet provision of dedicated foot care services for patients with inflammatory arthritis is variable [15–17]. Furthermore there is emerging evidence indicating foot care interventions are likely to be effective in patients with RA [18, 19]. Exploration of the patient experience of foot problems as a consequence of RA and access to foot care has received attention [20–22]. Overall these works indicate patients may not be receiving the timely and appropriate foot care. Whether or not patients receive adequate foot care depends not only upon the effects of their condition in general and on their feet in particular, but also in the way foot care services are provided and whether patients can gain access to them. Additionally access to and utilisation of health care in general is complex. A number of predisposing factors such as general characteristics (age, gender, social deprivation), clinical characteristics (nature of health condition acute or long term), experience and satisfaction of care received are reported to influence individuals in their decision to access health care of not [23].

Although foot problems are common in RA, their full impact, consequences and importance to patients and patient’s decisions to access foot care services have not been fully established. This current study therefore aimed to explore patients’ experience of foot problems and their decisions to access foot care services or not.

## Methods

### Study aims

1. Understand the impact of foot problems in patients with RA in relation to their personal experiences.
2. Discover patients’ reasons for accessing or not accessing foot care services.

### Participants

Patients with a consultant diagnosis of RA [24] and over 18 years of age were recruited from two rheumatology departments in the south west of England (UK). Patients were approached by a member of the rheumatology direct care team at both hospital sites, using the screening question “Do you have problems with your feet because of your RA?” and if they answered “Yes” were asked whether they had accessed foot care services (podiatry, orthotics and/or orthopaedics) since being diagnosed with RA. The rationale for the screening questions was to capture the patient experience of the topic of interest (experienced foot problems) and to provide the opportunity to discover patients’ reasons for accessing foot care or not. Those who expressed an interest to participate in the study were introduced to the researcher (OW) by a member of the rheumatology direct care team for further information. The researcher introduced herself as a research fellow not as a clinician. This was because disclosure of professional background has been reported to influence participants’ responses during qualitative interviews [25]. The researcher was not directly involved in the clinical care of any the patients approached to participate in the study. All patients expressing an interest (in the study) were provided with a patient information sheet with a reply slip (agreeing to be contacted by the researcher). All patients who returned the reply slip were considered for recruitment.

Local NHS research ethics committee approval was obtained (South West 4 Medical Regional Ethics Committee, 10/H01021/46) and written informed consent was obtained from all patients. Findings are reported using COREQ guidelines [26].

### Data collection

A purposive sampling strategy was adopted by means of a sampling frame [27] to capture a range of patient characteristics: age; gender; disease duration; accessed foot care services (podiatry, orthotics and/or orthopaedics) or not since being diagnosed with RA.

The interviews were conducted in clinical rooms at both hospital sites with only the participant and researcher present (except one interview where the participant brought her young child). A detailed account of the experience of foot problems, patients’ reasons for accessing foot care or not and experiences of care received were explored using an interview topic guide (Table 1). The interview topic guide was developed by the researcher based on a review of the literature; discussions with a research advisory group (consisting of patients, clinicians and academic supervisors) and a patient research partner (Table 2). The interviews were audio recorded and transcribed verbatim. The researcher checked the transcripts for accuracy by comparing all transcripts against audio recordings. All names and identities were then anonymised by the researcher. To maintain confidentiality all participants were allocated an identifier consisting of a code containing patient number.

**Table 1:** Interview topic guide

Questions and prompts	
1: Tell me the story about your feet?	
How important are your foot problems to you?	
Have you discussed your foot problems with anyone?	
2: What are your foot problems?	
Have your feet changed since developing RA?	
Has anyone examined your feet since developing RA?	
3: How do you manage your foot problems?	
Can you give an example?	
4: Have you had any experience of foot care services?	
If so, how did you access care?	
5: How much do your foot problems affect your activity?	
How do you feel about your foot problems affecting your activity levels?	
Are you able to drive, work, and take part in leisure activities?	
How do your foot problems affect the way you feel about things?	
6: Do they have an impact on your choice of shoes, clothes etc.?	
How does this make you feel?	
7: If we could make things better, do you have a wish list for foot care services?

**Table 2:** Study team characteristics

Team	Gender	Position	Years of rheumatology experience
OW	Female	Podiatrist/PhD student	15 years
ED	Female	Rheumatology psychology researcher	5 years
EQ	Female	Patient research partner	RA diagnosed >20 years
JK	Male	Academic rheumatologist	>30 years
SH	Female	Academic rheumatology nurse	>20 years

Prior to interview, participants provided demographic data (hospital site, age, gender), clinical data (arthritis medications, disease duration, disability (Health Assessment Questionnaire)) [28, 29], the patient global Numerical Rating Scale (NRS) from the Disease Activity Score (DAS) [30] and an NRS to measure the severity (magnitude) of foot problems (both 0–10, high-bad). These data provide a description of the general and clinical characteristics of the study participants. Additionally these descriptors of patients with RA are widely used in clinical research. Recruitment to the study continued until data saturation had been achieved, indicated by no new major issues emerging in three consecutive interviews [31]. The interviews lasted between 35 and 60 min.

### Analysis

Qualitative data were analysed using inductive thematic analysis (ITA), guided by the process described in Attride – Sterling and Braun and Clarke [32, 33]. Analysis was iterative and used constant comparison both within and between data sets. After reading and re-reading the data, initial codes were created. Codes with similar meaning were then grouped to form sub-themes, which in turn were clustered into organising themes, and finally organising were grouped to form global themes/underpinning themes to support the more abstract meaning of organising themes. In this iterative process themes identified in early interviews were explored in subsequent interviews and also applied to earlier data sets. The data handling package NVivo 8 (QSR International, Doncaster, Victoria, Australia) was utilised. The researcher (OW) analysed all transcripts. Three transcripts were also independently analysed by members of the study team (SH, ED and EQ) and overall emergent themes agreed by the whole study team. Descriptive statistics were used to describe the study participants’ general and clinical characteristics.

## Results

In total 27 patients with RA and self-reported foot problems (symptoms) were approached to participate in the study. Of these, 9 declined to participate - 2 indicated that they did not wish to attend for an additional hospital visit and 7 did not mention a reason. Of the 18 participants who expressed an interest in taking part in the study, 5 could not be contacted by telephone and did not respond to written invitations and 1 did not attend for the arranged one to one interview. The clinical and demographic descriptors of 12 patients who participated in the study are presented in Table 3. Data were collected between October 2010 and April 2011.

**Table 3:** Participant (patient) characteristics

ID	Gender	Age (years)	Disease duration (years)	Current medication	HAQ	Patient global	Foot global	Accessed foot care
1	Male	61	3	Biologics, DMARDs, GC	2.875	6	6	Yes
2	Female	62	2	DMARDs, GC	0.375	2	3	No
3	Male	39	2	DMARDs, GC, NSAIDs	0	1	1	No
4	Male	55	27	Biologics, DMARDs, NSAIDs	2.75	8	9	Yes
5	Female	61	23	DMARDS, GC, NSAIDs	2.75	7	7	Yes
6	Male	54	2	Biologics, DMARDS, GC, NSAIDs	2	8	9	No
7	Female	71	11	Biologics	0.375	7	1	No
8	Male	72	20	DMARDs, NSAIDs	1.87	9	5	Yes
9	Female	46	24	Biologics, DMARDs, NSAIDs	2.375	6	8	Yes
10	Female	29	5	GC	0	0	0	No
11	Female	55	7	DMARDs, NSAIDs	1.87	5	7	Yes
12	Female	69	18	DMARDs	1.75	7	8	Yes
Mean		56.2	12.0		1.56	5.5	5.3	
Range		(29–72)	(2–27)		(0–2.875)	(0–9)	(0–9)	

*Key*:

*ID* Patient identifier

*DMARDs* Disease-modifying anti-rheumatic drugs

*GC* Glucocorticoids

*NSAIDs* Non-steroidal anti-inflammatory drugs

*HAQ* Health Assessment Questionnaire score 0–3 (3 is most disabled)

Patient Global = Numerical rating scale 0 (very well) -10 (10 very badly)

Foot Global = Numerical rating scale 0 (no problem) -10 (10 severe problem)

**Fig. 1:**
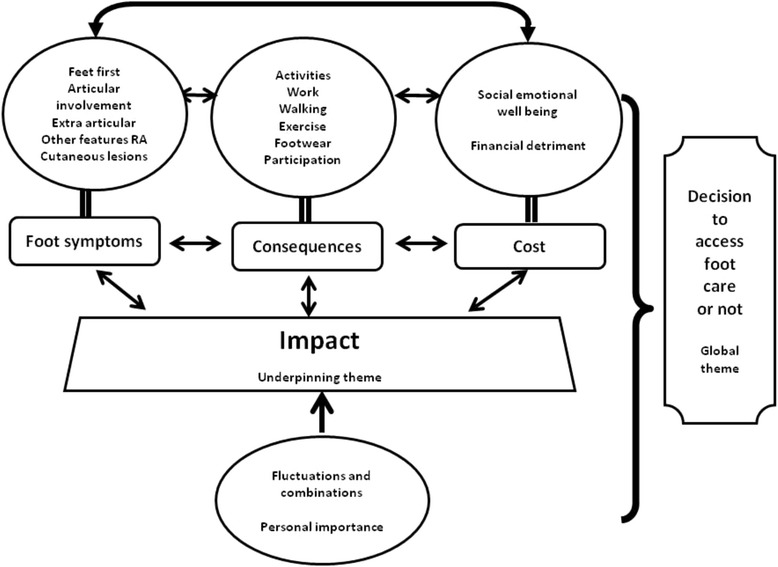
Overview of experience and impact of foot problems in RA

**Fig. 2:**
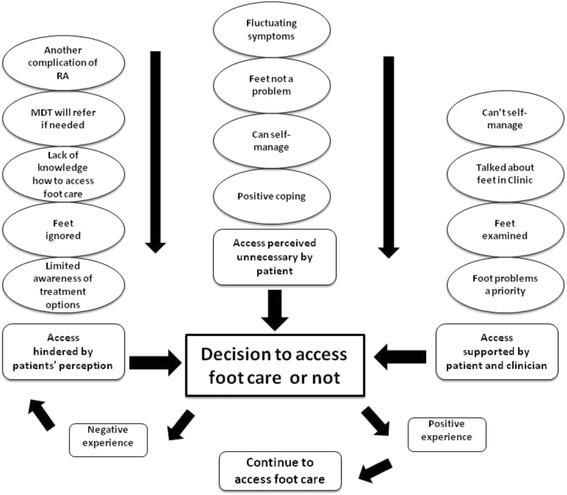
Decision to access foot care or not (Global theme)

Overall one underpinning theme (‘Impact’) and one global theme (‘Decision to access foot care or not’) emerged (Fig. 1). The underpinning ‘Impact’ theme was created from 159 codes, drawn together into 15 subthemes, and then 3 organising themes (‘Foot symptoms’; ‘Consequences’; and ‘Cost’). The global theme was created from 180 codes, drawn together into 14 subthemes and 3 organising themes: ‘Access hindered by patient perceptions’; ‘Access perceived unnecessary by patient’; and ‘Access supported by patient and clinician’ (Fig. 2). Detailed codes provided in Additional file 1: Tables S1 and S2.

### Underpinning theme: Impact of foot problems (Fig. 1)

‘Impact’ underpins the organising themes of foot symptoms, consequences and cost. Impact highlights that foot problems in RA may not be static or constant and appear to vary in severity. Varying levels of personal importance of the impact of foot problems were divulged. Whilst some participants discussed their foot problems at length but considered other clinical features of RA were greater personal issues, other participants considered feet to have high levels of personal importance as their foot problems were having significant consequences on their lives:

*“It’s actually my feet is what’s preventing me from getting around.” (Patient 8)*

#### Organising theme 1: *Foot symptoms*

The development of foot problems appeared to follow a variable and fluctuating clinical trajectory. Participants described a range of experiences with some recalling that the first symptoms of RA were in their feet. In contrast other participants described the evolvement of foot problems as a more gradual insidious clinical feature that varied in severity:

*“The first sign something was going on was my feet. The balls of the foot were really uncomfortable when I walked......” (Patient 3)*

In addition to experiencing pain and stiffness in the feet, particularly when walking, some participants highlighted that they were more conscious of their foot problems during a flare:

*“And when I get that [referring to a flare] I’m more conscious of my feet. When I’m not having a flare it’s only when I’ve walked too far or … stood for a long time. And then I become aware that my toes have become a bit stiff and my heels hurt.” (Patient 12)*

Participants reported a range of foot problems such as pain, stiffness, swelling, numbness, joint deformity, which patients felt led to cutaneous lesions (corn and callus formation, and toe nail pathologies):

*“I can see why I’ve got the callus just merely by the way when I stand. The nails have gone um … awful as well.” (Patient 7)*

#### Organising theme 2: *Consequences*

All participants discussed the issues their foot problems had on their ability to walk. The majority considered that the distance that they could now walk had reduced:

*“Some days I really can’t walk, the pain is so bad [referring to foot pain]. I’ve never broken a bone in my life. But if I had to imagine what a broken bone felt like, that’s what it feels like when I walk.” (Patient 11)*

Some participants considered that their foot problems had consequences on their ability to work. This was in relation to the nature of their occupations, such as standing for long periods of time, having to wear safety footwear or driving for long periods:

*“Couldn’t go in any of the workshops because of the safety, I couldn’t manage the safety shoes, they were too heavy for my feet.” (Patient 4)*

Exercise was considered a valued activity by some participants, not only for the benefits of general health and psychological wellbeing but also as part of their social life. Not being able to participate was a negative experience for some:

*“…and because I couldn’t do like the physical activity that I would usually do, like well a lot of running and stuff, that made me quite depressed.” (Patient 11)*

The consequences of foot problems in relation to footwear were discussed at length by all participants, including difficulties sourcing footwear that was comfortable, accommodating foot deformities and fluctuating symptoms. Finding aesthetically acceptable footwear was important and had consequences relating to choice of clothing, which was not gender-specific:

*“Awful because I was always one that wore high heels and you know and to wear these [referring to current footwear]. And they were … they looked so … clumpy you know. I was sort of begrudgingly wearing them. It was. It was… because they looked so old-fashioned.” (Patient 12)*

*“Most people get dressed from the top down; I get dressed from the bottom up.” (Patient 5)*

#### Organising theme 3: *Cost*

Participants referred to the cost of foot problems in terms of the effect on social and emotional well-being, with some describing the feelings of low mood and frustration from the loss of not being able to participate in valued activities, and embarrassment due to the presence of foot deformity and swelling:

*“One of the best things about football was going for a drink with the boys afterwards [.....] So I stopped going. Not only could I not play anymore but the loss of the social side, not seeing my mates and all that made me feel really low.” (Patient 4)*

*“Because my feet have got so wide and my toes have spaced out quite a bit. It doesn’t look very nice either. This ankle is permanently swollen and looks awful as well.” (Patient 8)*

Some participants described the cost of the consequences of their foot problems in terms of financial implications, including inability to continue with paid employment, and cost of footwear:

*“So I was falling over in the yard. And then what happened, the company had cameras put in everywhere and then I was sort of being sort of asked what the problem was with me shoes and with the boots they supplied.” (Patient 6)*

*“….my boots, the ones that I’ve worn all the way through the winter, but they haven’t been comfortable. But I couldn’t afford to buy another pair so I just had to make do.” (Patient 9)*

### Global theme: Decision to access foot care (Fig. 2)

The unique combination of each participant’s experience of foot problems, consequences and cost, underpinned by impact, led to decisions related to accessing foot care or not. Participants who had accessed foot care services prioritised their foot problems as an important health-care need. In contrast, other participants, whilst reporting foot problems and the consequences and impact of foot involvement as important issues, did not access foot care services.

#### Organising theme 4: *Access hindered by patient perceptions*

Participants acknowledged their foot problems were part of RA. Whilst some had confidence that the rheumatology clinical team would refer them for foot care if appropriate, most perceived that feet were often ignored by rheumatology clinicians and were not included in the assessment of disease status. The perception of the feet being ignored by the clinical team was then interpreted by some participants as being because there were no further care options available. However, some patients chose to ignore their own feet problems as they were not ready to make the necessary lifestyle changes:

*“What I tend to do is wait until I see [doctor’s name] and then he’s the one who would recommend me then [referral to foot care]. […] So I leave things like that to him and then he will refer me then. Because obviously he’s the expert.” (Patient 11)*

*“Because they’re [feet] not on any sort of thing. You know it’s not on any score thing is it? It sort of stops at the knee.” (Patient 6)*

*“I’ve got splints for my hands and wrist splints and all that you know that was done here [….] but it didn’t seem to be the same sort of thing for gravity on the uh …feet. (Patient 1)*

*“It’s not where I wanted to go [referred for prescribed footwear] it was another nail in the coffin … I didn’t want to go there.” (Patient 5)*

Participants had varying knowledge of foot care services in general terms. For some there was awareness that help was available if the need arose. In contrast others verbalised uncertainty about which foot care services would be available and how services could be accessed, and whether anything could be done to help:

*“Well I thought they were all private actually. I didn’t know you could access them [podiatrists] on the NHS.” (Patient 7)*

*“I wasn’t really sure that there was anything you could do to actually relieve the pain particularly in the feet. Other than to reduce the activity. I’ve just sort of plodded on assuming that there isn’t anything that could be done.” (Patient 11)*

#### Organising theme 5: *Access perceived unnecessary by patient*

As expected some patients’ foot problems were not a major concern. For others the fluctuating nature of foot symptoms influenced the decision not to access foot care, as they considered it was possible their symptoms would improve. The ability to self-manage was also provided as a reason not to access foot care, including applying heat and cold modalities and filing areas of callus:

*“I just very gently just do that with them [demonstrating using a foot file]. And I sort of try to keep the hard skin under control by doing that. Put cream on them, moisturiser. And then I give them a rub with my file.” (Patient 11)*

*“When my feet were playing up I’ve used liners [insoles]. You know the ones you can buy in the shops. They cost me but I didn’t mind. If they helped I didn’t mind how much they cost.” (Patient 5)*

#### Organising theme 6: *Access supported by patient and clinician*

The third organising theme ‘Access supported by patient and clinician’ illustrates the influence of feet being included in clinical consultations and foot problems being an important health care need for some patients. For their part, patients were sometimes pro-active in accessing their own foot care, for example when functional disability from RA reduced the ability to perform foot care:

*“Well I suppose because they were always checking (Patient 8 laughs) my feet it became fairly obvious that um … problems do occur with one’s hands and fingers and feet.” (Patient 8)*

*“When I had my hip done I couldn’t really get down to cut my toe nails and my husband said “oh I’ll do those for you” [....] “I said no it’s all right I’ll go to the clinic.” (Patient 5)*

Some patients were also pro-active in seeking clinician help if they felt feet were being ignored:

*“I took my shoes off and showed the … the woman [specialist nurse]. So that’s when she would have inspected, looked at them.” (Patient 10)*

For those who accessed foot care there was variation in the benefit of the care they received. Participants who perceived foot care received to be beneficial and therefore a positive experience, continued to access care. However, others had negative experiences whereby they felt that their foot health care needs were not fully addressed or the care they received was sub-optimal. As a consequence these participants discontinued accessing care:

*“As for going to the podiatrist then yes I suppose every six weeks or so then that does cost me but I think it’s worth the money really.” (Patient 5)*

*“Well they didn’t cut your nails, they didn’t do that. But they just really shaved all the skin off. But I really didn’t find them very … you know I could do that myself.” (Patient 12)*

## Discussion

The findings of this study provide a wide understanding of the ways in which foot problems impact on patients’ lives, and the reasons why patients do or do not access foot care services. Although it has previously been reported that foot problems affect patients’ ability to walk [1, 6], this study has highlighted that foot problems may also affect other activities such as ability to exercise, socialise and particularly to undertake paid employment. That RA in general can cause work disability has been reported [34–36] but this has been linked to general patient demographics, clinical variables and work related characteristics. Indeed, development of measures capturing information regarding patients’ ability to participate in paid employment has been proposed to be an important area for the research agenda [37]. This study highlights the importance of intrinsic foot problems as a cause of work related disability, not only through problems of mobility, but also for some patients their inability to comply with health and safety requirements. The overall contribution of foot problems to work disability within the broad picture of disability requires further elucidation.

The consequences of foot problems in relation to footwear were discussed by all patients. Some patients described difficulty in sourcing footwear that was comfortable, accommodated deformities and was aesthetically acceptable. Additionally, footwear influenced clothing choice which subsequently resulted in negative self-perceptions in relation to identity and body image. This confirms earlier work that footwear can be an important issue for patients [11, 12]. Previous work has tended to focus on the experience of female patients with RA but in this study footwear issues did not seem to be gender specific. Clearly, from previous studies but especially from the content of the interviews in the present study, the consequences of foot involvement in RA are complex and can impact on many aspects of patients’ lives, which would not be fully captured by functional status alone.

The Foot Impact Scale (FIS) [38] has been validated to quantify the impact of foot problems in RA and includes many of these concepts such as walking, footwear, activities, and participation. As the FIS is condition (RA) and anatomically specific it facilitates quantitative measurement of the impact of foot problems with confidence. Sanderson et al. [39], more recently postulated that the personal impact of RA may be influenced by patients’ ability to cope with a symptom, its perceived severity, and personal importance (Impact Triad). Almost all of the patients in this study described that their foot problems were severe at times, important and that they had difficulty self-managing. Therefore these data support Sanderson’s proposed theory of the Impact Triad. The importance and severity of foot problems to patients, their ability to cope with them and aspects of the impact of foot problems in relation work related disability, are not captured by the FIS, and these additional items related to foot problems have not previously been explored in patients with RA.

Factors influencing patients’ decisions to access foot care or not were complex. Patients who accessed foot care considered their foot problems to be an important health care need. In contrast, other patients reported that their foot problems were important issues but they had not accessed foot care. The issue of access to foot care, in particular access to podiatry services for patients with RA has received attention [21]. However, this earlier work focuses on factors influencing patients’ decisions to self-refer to foot care. Furthermore there is variation in access criteria to foot care in the UK, with access to some NHS services being restricted to clinician initiated referral only [40]. This current study highlights the importance of clinicians referring and/or recommendation as an additional factor influencing patients’ decisions to access foot care. The organising theme of ‘Access hindered by patients’ perceptions’ illustrates that some patients felt their feet had been ignored in clinical practice. Importantly, failure of clinicians to examine and/or discuss foot problems during consultations was considered by some patients to indicate that feet were unimportant, or no interventions were available because they assumed clinicians would instigate a referral if access to foot care were indicated. This finding supports similar conclusions in earlier work on foot problems [20, 22] and on fatigue [41]. The organising theme ‘Access hindered by patients’ perceptions’, related to limited awareness of treatment options and lack of knowledge of how to access care. However, some patients described how, despite current or past foot problems, access to foot care was considered unnecessary (‘Access perceived unnecessary by patient’) as their foot problems were not severe; they were able cope and could self-manage, adding further support to the Impact Triad theory [39]. The fluctuating nature of foot problems also influenced patients’ decisions to access foot care. It is possible that if symptoms persist for a relatively short time then patients may be unlikely to access foot care. Indeed, work conducted by Flurey et al. [42] in relation to help-seeking behaviours and flares in RA suggests patients will only access medical care when symptoms are overwhelming, when they are no longer able to cope and as a last resort.

Previous experience of foot care appeared to influence patients’ decisions whether or not to continue with utilising foot care services. Positive experiences of foot care appeared to favour continued utilisation of foot care services. Patients who perceived foot care received to be beneficial and therefore a positive experience, continued to access care. In contrast, previous negative experiences of foot care whereby patients felt that their foot health care needs were not fully addressed or the care they received was sub-optimal discontinued accessing foot care. These findings seem logical, and are supported by the Behavioural Model and Access to Health Care [23] in which continued utilisation is influenced by levels of customer (service user) satisfaction. If patients are dissatisfied with care received and/or do not consider interventions to be effective, the clinical benefits of foot care cannot be established. Both positive and negative experiences of foot care have been described in the literature [17, 20]. However, this current study is the first work to report the experience of foot care received influencing continuation and utilisation of foot care services by patients with RA.

Surveys conducted in rheumatology departments in both the UK and the southern hemisphere indicate the provision of dedicated foot services for patients with inflammatory arthritis to be variable and potentially inadequate [15, 16]. Additionally, there is considerable variation in the proportion of patients reported to have accessed foot care [3, 43, 44]. However, access to and the provision of foot care, and the professional remits of service providers, varies in different health care systems. Furthermore foot care services can also be provided in the independent (self-funding) sector. In this current study, patients who had accessed foot care services prioritised their foot problems as an important health care need. However, despite having foot problems some patients had not accessed care. Factors associated with decisions to access and utilise foot care services or not appear to be multifaceted and complex. Quantifying the extent to which these factors affect overall access to foot care in RA patients as a whole will help to inform the commissioning and provision of foot care for patients with RA.

### Strengths and limitations

The sample size in this study may be considered to limit transferability (or wider applicability) of findings to the wider RA patient population. Additionally all the study participants self-reported to have experienced foot problems at some time since being diagnosed with their RA. The issue of sample bias is therefore acknowledged. However, the sample was purposively diverse and data saturation was achieved [31]. Additionally an iterative approach was utilised during analyses in which emergence of new themes not previously addressed in the interview guide could be explored in subsequent interviews and in previous data sets [45]. Secondly, it is possible that patients in this study could have been describing the consequences and subsequent impact of more general features of RA. However, during the interviews the researcher used prompts to confirm patients were disclosing experiences relating to foot problems. The strength of this study is the elucidation of a rich description in relation to the broad spectrum of the experience, impact and care of RA foot problems from the patient’ perspective.

## Conclusion

This study further supports the body of literature that foot problems are important issues for patients and can substantially impact on many aspects their personal lives, including their ability to work. However, most of the patients in this study perceived that their feet were ignored in rheumatology consultations. Rheumatology and foot health clinicians need to recognise that foot problems in RA are common, can be variable and complex in presentation, are important issues for patients and should not be trivialised. Patients who had accessed foot care services prioritised their foot problems as an important health care need. However, despite having foot problems some patients had not accessed care. Further research is required to quantify the prevalence and impact of foot problems and access to foot care services in the population to inform the commissioning, organisation and delivery of foot care for patients with RA.

## Additional file

Additional file 1: Table S1. Coding tree overview impact and foot problems in RA. **Table S2.** Coding tree overview decision to access foot care. (DOCX 35 kb)

## Abbreviations

NHS: National Health Service
RA: Rheumatoid arthritis

## References

[1] van der Leeden M, Steultjens M, Ursum J (2008). Prevalence and cause of forefoot impairment and walking disability in the first eight years of rheumatoid arthritis. Arthritis Care Res.

[2] Michelson J, Easley M, Wigley FM, Hellmann D (1994). Foot and ankle problems in rheumatoid arthritis. Foot Ankle Int.

[3] Otter S, Lucas K, Springett K (2010). Foot pain in rheumatoid arthritis prevalence, risk factors and management: an epidemiological study. Clin Rheumatol.

[4] Spiegel JS, Spiegel TM (1982). Rheumatoid arthritis in the foot and ankle—diagnosis, pathology, and treatment: The relationship between foot and ankle deformity and disease duration in 50 patients. Foot Ankle Int.

[5] Turner DE, Helliwell PS, Siegel KL, Woodburn J (2008). Biomechanics of the foot in rheumatoid arthritis: identifying abnormal function and the factors associated with localised disease ‘impact’. Clin Biomech (Bristol, Avon).

[6] Grondal L, Tengstrand B, Nordmark B (2008). The foot: still the most important reason for walking incapacityin rheumatoid arthritis: distribution of symptomatic joints in 1000 RA patients. Acta Ortho.

[7] Wickman A, Pinzur M, Kadanoff R, Juknelis D (2004). Health-related quality of life for patients with rheumatoid arthritis foot involvement. Foot Ankle Int.

[8] Firth J, Hale C, Helliwell P (2008). The prevalence of foot ulceration in patients with rheumatoid arthritis. Arthritis Care Res.

[9] Siddle HJ, Redmond AC, Waxman R (2013). Debridement of painful forefoot plantar callosities in rheumatoid arthritis: the CARROT randomised controlled trial. Clin Rheum.

[10] Hooper L, Bowen C, Gates L (2012). Prognostic indicators of foot‐related disability in patients with rheumatoid arthritis: Results of a prospective three‐year study. Arthritis Care Res.

[11] Naidoo S, Anderson S, Mills J et al. “I could cry, the amount of shoes I can’t get into”: a qualitative exploration of the factors that influence retail footwear selection in women with rheumatoid arthritis. J Foot Ankle Res. 2011;doi:10.1186/1757-1146-4-21. 10.1186/1757-1146-4-21 PMC3166890 21794134

[12] Goodacre LJ, Candy FJ (2011). ‘If I didn’t have RA I wouldn’t give them house room’: the relationship between RA, footwear and clothing choices. Rheumatology (Oxford).

[13] Semple R, Newcombe LW, Finlayson GL (2009). The FOOTSTEP self‐management foot care programme: Are rheumatoid arthritis patients physically able to participate?. Musculoskeletal Care.

[14] Luqmani R, Hennell S, Estrach C (2006). British Society for Rheumatology and British Health Professionals in Rheumatology Guideline for the Management of Rheumatoid Arthritis (The first 2 years). Rheumatology (Oxford).

[15] Redmond A, Waxman R, Helliwell P (2006). Provision of foot health services in rheumatology in the UK. Rheumatology (Oxford).

[16] Rome K, Gow PJ, Dalbeth N, Chapman JM. Clinical audit of foot problems in patients with rheumatoid arthritis treated at Counties Manukau District Health Board, Auckland, New Zealand. J Foot Ankle Res. 2009;doi:10.1186/1757-1146-2-16. 10.1186/1757-1146-2-16 PMC2685775 19442310

[17] Hendry GJ, Gibson KA, Pile K et al. “They just scraped off the calluses”: a mixed methods exploration of foot care access and provision for people with rheumatoid arthritis in south-western Sydney, Australia. J Foot Ankle Res. 2013;doi:10.1186/1757-1146-6-3. 10.1186/1757-1146-6-34 PMC3751079 23938103

[18] Farrow SJ, Kingsley GH, Scott DL (2005). Interventions for foot disease in rheumatoid arthritis: a systematic review. Arthritis Care Res.

[19] Hennessy K, Woodburn J, Steultjens MP (2012). Custom foot orthoses for rheumatoid arthritis: a systematic review. Arthritis Care Res.

[20] Williams A, Graham A (2012). ‘My feet–visible, but ignored…’A qualitative study of foot care for people with rheumatoid arthritis. Clin Rehabil.

[21] Blake A, Mandy PJ, Stew G (2013). Factors influencing the patient with rheumatoid arthritis in their decision to seek podiatry. Musculoskeletal Care.

[22] de Souza S, Williams R, Lempp H. Patient and clinician views on the quality of foot health care for rheumatoid arthritis outpatients: a mixed methods service evaluation. J Foot Ankle Res. 2016;doi:10.1186/s13047-015-0133-2. 10.1186/s13047-015-0133-2 PMC4702354 26740821

[23] Andersen R (1995). Revisiting the behavioral model and access to medical care: does it matter?. J Health Soc Behav.

[24] Arnett FC, Edworthy SM, Bloch DA (1988). The American Rheumatism Association 1987 revised criteria for the classification of rheumatoid arthritis. Arthritis Rheum.

[25] Richards H, Emslie C (2000). The ‘doctor’or the ‘girl from the University’? Considering the influence of professional roles on qualitative interviewing. Fam Prac.

[26] Tong A, Sainsbury P, Craig J (2007). Consolidated criteria for reporting qualitative research (COREQ): a 32-item checklist for interviews and focus groups. Int J Qual Health Care.

[27] Marshall MN (1996). Sampling for qualitative research. Fam Pract.

[28] Fries J, Spitz P, Kraines R, Holman H (1980). Measurement of patient outcome in arthritis. Arthritis Rheum.

[29] Kirwan J, Reeback J (1996). Stanford Health Assessment Questionnaire modified to assess disability in British patients with rheumatoid arthritis. Rheumatology (Oxford).

[30] van der Heijde DM, van't Hof M, van Riel P, van de Putte LB (1993). Development of a disease activity score based on judgment in clinical practice by rheumatologists. J Rheumatol.

[31] Guest G, Bunce A, Johnson L (2006). How many interviews are enough? An experiment with data saturation and variability. Field Methods.

[32] Attride-Sterling J (2001). Thematic networks: an analytical tool for qualitative research. Qual Res.

[33] Braun V, Clark V (2006). Using thematic analysis in psycology. Qual Res Psycol.

[34] Barrett E, Scott D, Wiles NJ, Symmons D (2000). The impact of rheumatoid arthritis on employment status in the early years of disease: a UK community‐based study. Rheumatology (Oxford).

[35] Verstappen SM, Bijlsma JW, Verkleij H (2004). Overview of work disability in rheumatoid arthritis patients as observed in cross‐sectional and longitudinal surveys. Arthritis Care Res.

[36] Olofsson T, Petersson I, Eriksson J (2014). Predictors of work disability during the first 3 years after diagnosis in a national rheumatoid arthritis inception cohort. Ann Rheum Dis.

[37] Walker-Bone K, Black C (2016). The importance of work participation as an outcome in rheumatology. Rheumatology (Oxford).

[38] Helliwell P, Reay N, Gilworth G (2005). Development of a foot impact scale for rheumatoid arthritis. Arthritis Care Res.

[39] Sanderson T, Hewlett S, Flurey C (2011). The impact triad (severity, importance, self-management) as a method of enhancing measurement of personal life impact of rheumatic diseases. J Rheumatol.

[40] England NHS (2015). Improving the quality of orthotics in England.

[41] Hewlett S, Cockshott Z, Byron M (2005). Patients’ perceptions of fatigue in rheumatoid arthritis: overwhelming, uncontrollable, ignored. Arthritis Care Res.

[42] Flurey C, Morris M, Richards P (2014). It’s like a juggling act: rheumatoid arthritis patient perspectives on daily life and flare while on current treatment regimes. Rheumatology (Oxford).

[43] Backhouse MR, Keenan AM, Hensor EM, Young A, James D, Dixey JR (2011). Use of conservative and surgical foot care in an inception cohort of patients with rheumatoid arthritis. Rheumatology (Oxford).

[44] Marsman AF, Dahmen R, Roorda LD (2013). Foot‐related health care use in patients with rheumatoid arthritis in an outpatient secondary care center for rheumatology and rehabilitation in The Netherlands: a cohort study with a maximum of fifteen years of followup. Arthritis Care Res.

[45] Lewis J, Ritchie J, Ormston R, Morrell G, Ritchie J, Lewis J, McNaughton Nicholls C, Ormston R (2013). Generalising from qualitative research. Qualitative research practice: A guide for social science students and researchers.

