# Supplementary material for: The experience of foot problems and decisions to access foot care in patients with rheumatoid arthritis: a qualitative study
Source: J Foot Ankle Res. 2017 Jan 25;10:4. doi: 10.1186/s13047-017-0188-3 (PMC5264322; doi:10.1186/s13047-017-0188-3)
Supplement: Additional file 1: Table S1. — Coding tree overview impact and foot problems in RA. Table S2. Coding tree overview decision to access foot care. (DOCX 35 kb) [file 13047_2017_188_MOESM1_ESM.docx]

## Supplementary data: Table S1 Coding tree overview impact and foot problems in RA

| **Codes** | **Subthemes** | **Organising themes** | **Under pinning theme** | **Global theme** |
| --- | --- | --- | --- | --- |
| 1^st^ foot symptoms | Feet first | Foot symptoms | Impact | Decision to access foot care of not |
| Balls of feet painful |  |  |  |  |
| Couldn’t walk |  |  |  |  |
| Feet and toes stiff |  |  |  |  |
| Feet changed shape |  |  |  |  |
| Feet painful at rest |  |  |  |  |
| Feet sensitive |  |  |  |  |
| Feet were bad |  |  |  |  |
| Feet were really uncomfortable |  |  |  |  |
| Foot problems came later |  |  |  |  |
| Foot problems didn’t go away |  |  |  |  |
| Pain started in feet |  |  |  |  |
| Started (RA) in feet |  |  |  |  |
| Surprised by foot involvement |  |  |  |  |
| Swelling in feet |  |  |  |  |
| Ankle problems | Articular involvement |  |  |  |
| Bunions |  |  |  |  |
| Current foot problems |  |  |  |  |
| Fallen arches |  |  |  |  |
| Feet ache |  |  |  |  |
| Feet always hurting |  |  |  |  |
| Feet painful at rest |  |  |  |  |
| Feet painful in the morning |  |  |  |  |
| Feet throb |  |  |  |  |
| Feet uncomfortable |  |  |  |  |
| Foot deformity |  |  |  |  |
| Foot pain |  |  |  |  |
| Foot pain crippling |  |  |  |  |
| Consequences of foot pain |  |  |  |  |
| Misshapen toes |  |  |  |  |
| No foot deformity |  |  |  |  |
| Pain like a broken bone |  |  |  |  |
| Secondary OA |  |  |  |  |
| Burase | Extra-articular |  |  |  |
| Feet burn |  |  |  |  |
| Nodules |  |  |  |  |
| Numbness in feet |  |  |  |  |
| Swelling in feet |  |  |  |  |
| Advise for patients newly diagnosed with RA | Other features RA |  |  |  |
| Concerns about the future |  |  |  |  |
| Fatigue |  |  |  |  |
| Feet part of whole RA |  |  |  |  |
| General RA symptoms |  |  |  |  |
| Hand problems |  |  |  |  |
| Morning stiffness |  |  |  |  |
| Other joints |  |  |  |  |
| Pain |  |  |  |  |
| RA and activities of daily living |  |  |  |  |
| RA and pregnancy |  |  |  |  |
| RA and relationships with others |  |  |  |  |
| Blisters | Cutaneous lesion |  |  |  |
| Calluses / hard skin |  |  |  |  |
| Corns / stone bruises |  |  |  |  |
| Ingrown toe nails |  |  |  |  |
| Nails changed |  |  |  |  |
| Skin infections |  |  |  |  |
| Skin problems |  |  |  |  |
| Driving | Activities | Consequences |  |  |
| Can’t brake suddenly |  |  |  |  |
| Can’t depress clutch |  |  |  |  |
| Can’t stand for long |  |  |  |  |
| Reliance on others |  |  |  |  |
| Social life |  |  |  |  |
| Couldn’t stand for long | Work |  |  |  |
| Couldn’t wear safety boots |  |  |  |  |
| Falls at work |  |  |  |  |
| Feet and work |  |  |  |  |
| Had to stop work |  |  |  |  |
| Receiving financial assistance |  |  |  |  |
| Retired |  |  |  |  |
| Work adaptations |  |  |  |  |
| Work not an issue |  |  |  |  |
| Can’t walk around the shops | Walking |  |  |  |
| Can’t walk as far as I used to |  |  |  |  |
| Can’t walk far |  |  |  |  |
| Can’t walk up hills |  |  |  |  |
| Feet stopping me getting around |  |  |  |  |
| Feet stopping me walking |  |  |  |  |
| Have to plan ahead |  |  |  |  |
| Have to walk more slowly |  |  |  |  |
| Painful to walk |  |  |  |  |
| Steps difficult |  |  |  |  |
| Walking on bag of bones |  |  |  |  |
| Walking on glass |  |  |  |  |
| Walking on pebbles |  |  |  |  |
| Walking on uneven surfaces |  |  |  |  |
| Benefits of exercise | Exercise |  |  |  |
| Can’t exercise because of feet |  |  |  |  |
| Cycling and feet |  |  |  |  |
| Exercise and feet |  |  |  |  |
| Exercise and mood |  |  |  |  |
| Feel depressed when can’t exercise |  |  |  |  |
| Had to look for other types of activity |  |  |  |  |
| Choice of clothes | Footwear |  |  |  |
| Comfortable footwear |  |  |  |  |
| Difficult to get shoes |  |  |  |  |
| Difficult to get shoes to fit |  |  |  |  |
| Femininity and masculinity |  |  |  |  |
| Fluctuating comfort of shoes |  |  |  |  |
| Foot deformity and footwear |  |  |  |  |
| Footwear and body image |  |  |  |  |
| Footwear and gender |  |  |  |  |
| Footwear and identity |  |  |  |  |
| Footwear and walking |  |  |  |  |
| Footwear and work |  |  |  |  |
| Footwear soles |  |  |  |  |
| Given shoes away |  |  |  |  |
| Had to stop wearing certain shoes |  |  |  |  |
| Footwear fastenings |  |  |  |  |
| Importance of shoes fitting |  |  |  |  |
| Improvements to footwear |  |  |  |  |
| Looking for shoes disheartening |  |  |  |  |
| Need different types of shoes |  |  |  |  |
| Not ready to get rid of shoes |  |  |  |  |
| Visual appearance of footwear |  |  |  |  |
| Can’t dance anymore | Participation |  |  |  |
| Can’t do what my friends do |  |  |  |  |
| Can’t participate in team sports |  |  |  |  |
| Don’t go out any more |  |  |  |  |
| Don’t the things I used to |  |  |  |  |
| Everyone else having a conversation |  |  |  |  |
| Isolation |  |  |  |  |
| Missing out |  |  |  |  |
| Can’t be a good parent | Social and emotional well being | Cost |  |  |
| Don’t like situation |  |  |  |  |
| Embarrassed about appearance of feet |  |  |  |  |
| Embarrassed to show feet |  |  |  |  |
| Feet frustrating |  |  |  |  |
| Feet make me look old |  |  |  |  |
| Foot problems annoying |  |  |  |  |
| Holding everyone back |  |  |  |  |
| Makes me snappy |  |  |  |  |
| Reliance on others |  |  |  |  |
| Cost of insoles | Financial detriment |  |  |  |
| Cost of shoes |  |  |  |  |
| Had to change type of car |  |  |  |  |
| Had to give up work |  |  |  |  |
| Money spent on feet |  |  |  |  |
| Comes and goes | Fluctuations and combinations |  |  |  |
| Evolving foot symptoms |  |  |  |  |
| Feet and arthritis medications |  |  |  |  |
| Feet and flare |  |  |  |  |
| Fluctuating foot symptoms |  |  |  |  |
| Foot problems unpredictable |  |  |  |  |
| It varies (foot symptoms) |  |  |  |  |
| Today is a good day |  |  |  |  |
| Concern about foot problems developing | Personal importance |  |  |  |
| Current foot symptoms big problems |  |  |  |  |
| Feel trapped |  |  |  |  |
| Feet biggest problem of RA |  |  |  |  |
| Feet effect everything |  |  |  |  |
| Feet not a problem |  |  |  |  |
| Feet ok at the moment |  |  |  |  |
| Have to get on with it |  |  |  |  |
| Importance of foot problems |  |  |  |  |
| Importance of looking after feet |  |  |  |  |
| Individual symptoms and need |  |  |  |  |
| Something I have to put up with |  |  |  |  |
| Worst bit of me |  |  |  |  |

## Supplementary data: Table S2 Coding tree decision to access foot care

| **Codes** | **Subthemes** | **Organising themes** | **Global theme** |
| --- | --- | --- | --- |
| Acceptance | Another complication of RA | Access hindered by patient’s perception | Decision to access foot care or not |
| Been lucky |  |  |  |
| Feet minor part of RA |  |  |  |
| Fluctuating symptoms |  |  |  |
| Foot problems due to RA |  |  |  |
| Foot problems unpredictable |  |  |  |
| Least of my worries |  |  |  |
| Loss |  |  |  |
| More appointments |  |  |  |
| Other priorities |  |  |  |
| Other RA symptoms a priority |  |  |  |
| Terrible disease |  |  |  |
| Bombarded with information | MDT will refer if needed |  |  |
| Changed my life |  |  |  |
| Clinicians very helpful |  |  |  |
| Doctor knows best |  |  |  |
| Doctor organised education sessions |  |  |  |
| Doctor organises what I need |  |  |  |
| Doctor very good |  |  |  |
| Doctor very thorough |  |  |  |
| Doctor will refer if I need it |  |  |  |
| Don’t want RA care anywhere else |  |  |  |
| Focus on development and research |  |  |  |
| Grateful for treatment |  |  |  |
| Lots of support |  |  |  |
| Miracle cure |  |  |  |
| Physiotherapy helped |  |  |  |
| RA well managed |  |  |  |
| Referred to OT |  |  |  |
| Very grateful for care |  |  |  |
| Can’t arrange an appointment myself | Lack of knowledge how to access foot care |  |  |
| Didn’t know could get help |  |  |  |
| Doctor has to organised it |  |  |  |
| Doctor hasn’t arranged it |  |  |  |
| Don’t know how to get an appointment |  |  |  |
| Don’t know how to get to see a podiatrist |  |  |  |
| Don’t know what an orthotics is |  |  |  |
| Lack of knowledge of service location |  |  |  |
| Nobody has suggested |  |  |  |
| Thought they were all private |  |  |  |
| Another nail in the coffin | Feet ignored |  |  |
| Been recommended but resisting |  |  |  |
| Clinicians focus on hands |  |  |  |
| Clinicians focus on large joints |  |  |  |
| Didn’t associate foot problems with RA |  |  |  |
| Don’t want prescribed shoes |  |  |  |
| Don’t want surgery |  |  |  |
| Feet not examined |  |  |  |
| Feet not examined regularly |  |  |  |
| Feet not obvious |  |  |  |
| Feet not on any questionnaires |  |  |  |
| Feet not on assessments |  |  |  |
| Frankenstein boots |  |  |  |
| Limited clinical experience of assessing feet |  |  |  |
| Limited clinical experience of foot problems |  |  |  |
| No advise about foot problems |  |  |  |
| Not asked about feet |  |  |  |
| Not ready for prescribed shoes |  |  |  |
| Not ready for surgery |  |  |  |
| Not talked about feet |  |  |  |
| RA only affects joints |  |  |  |
| Don’t know what they do | Limited awareness of treatment options |  |  |
| Don’t know what will help |  |  |  |
| Everyone’s different |  |  |  |
| It’s an operation or nothing |  |  |  |
| Just cut toe nails |  |  |  |
| Just plodded on |  |  |  |
| Need a magic wand |  |  |  |
| No advise about treatment options |  |  |  |
| Nothing can be done |  |  |  |
| Podiatrist only cut corns |  |  |  |
| Comes and goes | Fluctuating symptoms | Access perceived unnecessary by patient |  |
| Enough pain for one day |  |  |  |
| Feet came later |  |  |  |
| Feet more painful in the morning |  |  |  |
| Feet bad today |  |  |  |
| Pain usually somewhere |  |  |  |
| Pain not everyday |  |  |  |
| Some days I can’t walk |  |  |  |
| Some days ok |  |  |  |
| Sometimes can’t get shoes |  |  |  |
| Sometimes need crutches |  |  |  |
| Swelling (feet) varies |  |  |  |
| Today is a good day |  |  |  |
| Varying degrees of pain |  |  |  |
| Worried will end up in wheelchair |  |  |  |
| Worse (feet) in the morning |  |  |  |
| Feet just uncomfortable | Feet not a problem |  |  |
| Feet not a big problem |  |  |  |
| Feet not changed shape |  |  |  |
| Feet ok today |  |  |  |
| Foot problems calmed down |  |  |  |
| Foot problems improved when RA controlled |  |  |  |
| Injections helped |  |  |  |
| Never had calluses |  |  |  |
| Not much of an issue (feet) |  |  |  |
| Bought my own insoles | Can self-manage |  |  |
| Cream my feet |  |  |  |
| Cycling helps foot pain |  |  |  |
| Experiment with shoes |  |  |  |
| Family help me (with feet) |  |  |  |
| Good shoes important |  |  |  |
| Know how to look after my feet |  |  |  |
| Know which shoes I need |  |  |  |
| Massage my feet |  |  |  |
| Old lady shoes |  |  |  |
| Take a second pair of shoes |  |  |  |
| Use a foot file |  |  |  |
| Use cold |  |  |  |
| Use heat |  |  |  |
| Wear comfortable shoes |  |  |  |
| Could be worse | Positive coping |  |  |
| I’m lucky |  |  |  |
| Meet them half way |  |  |  |
| Not as bad as others |  |  |  |
| Not on my own |  |  |  |
| You’ve got to try |  |  |  |
| Can’t manage feet because of hands | Can’t self-manage | Access supported by patient and clinician |  |
| Can’t reach feet |  |  |  |
| Can’t tie shoe laces because of hands |  |  |  |
| Don’t know which insole to try |  |  |  |
| Foot care helped others |  |  |  |
| Have to rely on others |  |  |  |
| Husband told me to go and see someone |  |  |  |
| Other joint problems |  |  |  |
| Wife won’t cut toe nails |  |  |  |
| Always tell the doctor feet bad | Talked about feet in clinic |  |  |
| Clinician asked about feet |  |  |  |
| Foot pain severe |  |  |  |
| Others told me insoles helped |  |  |  |
| The bunion was hurting |  |  |  |
| Told foot problems due to RA |  |  |  |
| Told nurse about feet |  |  |  |
| Wife told me to tell them about ankles |  |  |  |
| Bare foot examinations | Feet examined |  |  |
| Feet examined at diagnosis |  |  |  |
| Had scan of feet |  |  |  |
| Had x-rays of feet |  |  |  |
| Regular foot examination |  |  |  |
| Calluses painful | Foot problems a priority |  |  |
| Can’t cut toe nails |  |  |  |
| Can’t do what I want to do |  |  |  |
| Can’t get shoes to fit |  |  |  |
| Can’t stand for long |  |  |  |
| Can’t walk |  |  |  |
| Can’t walk round the shops |  |  |  |
| Don’t want them (feet) to get bad |  |  |  |
| Feet so painful |  |  |  |
| Feet stopping me being a parent |  |  |  |
| Feet stopping me doing things |  |  |  |
| Feet stopping me getting about |  |  |  |
| Feet stopping me working |  |  |  |
| Foot problems getting worse |  |  |  |
| Need to do something about feet |  |  |  |
| Suffer if I walk to far |  |  |  |
| Took shoes off and showed the nurse |  |  |  |
| Trapped in the house |  |  |  |
| Walk more slowly |  |  |  |
| Feet worst part of me |  |  |  |
| Can wear nice shoes again | Positive experience | Continue to access foot care |  |
| Due for new insoles |  |  |  |
| Feet better after surgery |  |  |  |
| Foot problems improved |  |  |  |
| It helps |  |  |  |
| Lucky to have insoles made |  |  |  |
| Podiatrist gives advice |  |  |  |
| Podiatrist keeps an eye on feet |  |  |  |
| Podiatrist treats problems as they occur |  |  |  |
| Worth the money |  |  |  |
| Insoles didn’t help | Negative experience |  |  |
| Couldn’t afford to keep paying for podiatry |  |  |  |
| Couldn’t be bothered to argue about shoes |  |  |  |
| Couldn’t get an appointment when needed |  |  |  |
| Couldn’t get insoles in shoes |  |  |  |
| Difficult to get appointments |  |  |  |
| Disappointed with podiatry |  |  |  |
| Didn’t cut toe nails |  |  |  |
| Hated the shoes |  |  |  |
| Long time to recover after surgery |  |  |  |
| Nice (podiatrist) but inexperienced |  |  |  |
| No advice given |  |  |  |
| No follow up care |  |  |  |
| Not much benefit from podiatry |  |  |  |
| Only shaved hard skin off |  |  |  |
| Operation didn’t help |  |  |  |
| Poor follow up care |  |  |  |
| Post surgery complications |  |  |  |
| Shoes didn’t fit |  |  |  |
| Shoes looked awful |  |  |  |
| Shoes too heavy |  |  |  |
| Waited a long time to be seen |  |  |  |

## Supplementary data: Table S2 Coding tree decision to access foot care

| **Codes** | **Subthemes** | **Organising themes** | **Global theme** |
| --- | --- | --- | --- |
| Acceptance | Another complication of RA | Access hindered by patient’s perception | Decision to access foot care or not |
| Been lucky |  |  |  |
| Feet minor part of RA |  |  |  |
| Fluctuating symptoms |  |  |  |
| Foot problems due to RA |  |  |  |
| Foot problems unpredictable |  |  |  |
| Least of my worries |  |  |  |
| Loss |  |  |  |
| More appointments |  |  |  |
| Other priorities |  |  |  |
| Other RA symptoms a priority |  |  |  |
| Terrible disease |  |  |  |
| Bombarded with information | MDT will refer if needed |  |  |
| Changed my life |  |  |  |
| Clinicians very helpful |  |  |  |
| Doctor knows best |  |  |  |
| Doctor organised education sessions |  |  |  |
| Doctor organises what I need |  |  |  |
| Doctor very good |  |  |  |
| Doctor very thorough |  |  |  |
| Doctor will refer if I need it |  |  |  |
| Don’t want RA care anywhere else |  |  |  |
| Focus on development and research |  |  |  |
| Grateful for treatment |  |  |  |
| Lots of support |  |  |  |
| Miracle cure |  |  |  |
| Physiotherapy helped |  |  |  |
| RA well managed |  |  |  |
| Referred to OT |  |  |  |
| Very grateful for care |  |  |  |
| Can’t arrange an appointment myself | Lack of knowledge how to access foot care |  |  |
| Didn’t know could get help |  |  |  |
| Doctor has to organised it |  |  |  |
| Doctor hasn’t arranged it |  |  |  |
| Don’t know how to get an appointment |  |  |  |
| Don’t know how to get to see a podiatrist |  |  |  |
| Don’t know what an orthotics is |  |  |  |
| Lack of knowledge of service location |  |  |  |
| Nobody has suggested |  |  |  |
| Thought they were all private |  |  |  |
| Another nail in the coffin | Feet ignored |  |  |
| Been recommended but resisting |  |  |  |
| Clinicians focus on hands |  |  |  |
| Clinicians focus on large joints |  |  |  |
| Didn’t associate foot problems with RA |  |  |  |
| Don’t want prescribed shoes |  |  |  |
| Don’t want surgery |  |  |  |
| Feet not examined |  |  |  |
| Feet not examined regularly |  |  |  |
| Feet not obvious |  |  |  |
| Feet not on any questionnaires |  |  |  |
| Feet not on assessments |  |  |  |
| Frankenstein boots |  |  |  |
| Limited clinical experience of assessing feet |  |  |  |
| Limited clinical experience of foot problems |  |  |  |
| No advise about foot problems |  |  |  |
| Not asked about feet |  |  |  |
| Not ready for prescribed shoes |  |  |  |
| Not ready for surgery |  |  |  |
| Not talked about feet |  |  |  |
| RA only affects joints |  |  |  |
| Don’t know what they do | Limited awareness of treatment options |  |  |
| Don’t know what will help |  |  |  |
| Everyone’s different |  |  |  |
| It’s an operation or nothing |  |  |  |
| Just cut toe nails |  |  |  |
| Just plodded on |  |  |  |
| Need a magic wand |  |  |  |
| No advise about treatment options |  |  |  |
| Nothing can be done |  |  |  |
| Podiatrist only cut corns |  |  |  |
| Comes and goes | Fluctuating symptoms | Access perceived unnecessary by patient |  |
| Enough pain for one day |  |  |  |
| Feet came later |  |  |  |
| Feet more painful in the morning |  |  |  |
| Feet bad today |  |  |  |
| Pain usually somewhere |  |  |  |
| Pain not everyday |  |  |  |
| Some days I can’t walk |  |  |  |
| Some days ok |  |  |  |
| Sometimes can’t get shoes |  |  |  |
| Sometimes need crutches |  |  |  |
| Swelling (feet) varies |  |  |  |
| Today is a good day |  |  |  |
| Varying degrees of pain |  |  |  |
| Worried will end up in wheelchair |  |  |  |
| Worse (feet) in the morning |  |  |  |
| Feet just uncomfortable | Feet not a problem |  |  |
| Feet not a big problem |  |  |  |
| Feet not changed shape |  |  |  |
| Feet ok today |  |  |  |
| Foot problems calmed down |  |  |  |
| Foot problems improved when RA controlled |  |  |  |
| Injections helped |  |  |  |
| Never had calluses |  |  |  |
| Not much of an issue (feet) |  |  |  |
| Bought my own insoles | Can self-manage |  |  |
| Cream my feet |  |  |  |
| Cycling helps foot pain |  |  |  |
| Experiment with shoes |  |  |  |
| Family help me (with feet) |  |  |  |
| Good shoes important |  |  |  |
| Know how to look after my feet |  |  |  |
| Know which shoes I need |  |  |  |
| Massage my feet |  |  |  |
| Old lady shoes |  |  |  |
| Take a second pair of shoes |  |  |  |
| Use a foot file |  |  |  |
| Use cold |  |  |  |
| Use heat |  |  |  |
| Wear comfortable shoes |  |  |  |
| Could be worse | Positive coping |  |  |
| I’m lucky |  |  |  |
| Meet them half way |  |  |  |
| Not as bad as others |  |  |  |
| Not on my own |  |  |  |
| You’ve got to try |  |  |  |
| Can’t manage feet because of hands | Can’t self-manage | Access supported by patient and clinician |  |
| Can’t reach feet |  |  |  |
| Can’t tie shoe laces because of hands |  |  |  |
| Don’t know which insole to try |  |  |  |
| Foot care helped others |  |  |  |
| Have to rely on others |  |  |  |
| Husband told me to go and see someone |  |  |  |
| Other joint problems |  |  |  |
| Wife won’t cut toe nails |  |  |  |
| Always tell the doctor feet bad | Talked about feet in clinic |  |  |
| Clinician asked about feet |  |  |  |
| Foot pain severe |  |  |  |
| Others told me insoles helped |  |  |  |
| The bunion was hurting |  |  |  |
| Told foot problems due to RA |  |  |  |
| Told nurse about feet |  |  |  |
| Wife told me to tell them about ankles |  |  |  |
| Bare foot examinations | Feet examined |  |  |
| Feet examined at diagnosis |  |  |  |
| Had scan of feet |  |  |  |
| Had x-rays of feet |  |  |  |
| Regular foot examination |  |  |  |
| Calluses painful | Foot problems a priority |  |  |
| Can’t cut toe nails |  |  |  |
| Can’t do what I want to do |  |  |  |
| Can’t get shoes to fit |  |  |  |
| Can’t stand for long |  |  |  |
| Can’t walk |  |  |  |
| Can’t walk round the shops |  |  |  |
| Don’t want them (feet) to get bad |  |  |  |
| Feet so painful |  |  |  |
| Feet stopping me being a parent |  |  |  |
| Feet stopping me doing things |  |  |  |
| Feet stopping me getting about |  |  |  |
| Feet stopping me working |  |  |  |
| Foot problems getting worse |  |  |  |
| Need to do something about feet |  |  |  |
| Suffer if I walk to far |  |  |  |
| Took shoes off and showed the nurse |  |  |  |
| Trapped in the house |  |  |  |
| Walk more slowly |  |  |  |
| Feet worst part of me |  |  |  |
| Can wear nice shoes again | Positive experience | Continue to access foot care |  |
| Due for new insoles |  |  |  |
| Feet better after surgery |  |  |  |
| Foot problems improved |  |  |  |
| It helps |  |  |  |
| Lucky to have insoles made |  |  |  |
| Podiatrist gives advice |  |  |  |
| Podiatrist keeps an eye on feet |  |  |  |
| Podiatrist treats problems as they occur |  |  |  |
| Worth the money |  |  |  |
| Insoles didn’t help | Negative experience |  |  |
| Couldn’t afford to keep paying for podiatry |  |  |  |
| Couldn’t be bothered to argue about shoes |  |  |  |
| Couldn’t get an appointment when needed |  |  |  |
| Couldn’t get insoles in shoes |  |  |  |
| Difficult to get appointments |  |  |  |
| Disappointed with podiatry |  |  |  |
| Didn’t cut toe nails |  |  |  |
| Hated the shoes |  |  |  |
| Long time to recover after surgery |  |  |  |
| Nice (podiatrist) but inexperienced |  |  |  |
| No advice given |  |  |  |
| No follow up care |  |  |  |
| Not much benefit from podiatry |  |  |  |
| Only shaved hard skin off |  |  |  |
| Operation didn’t help |  |  |  |
| Poor follow up care |  |  |  |
| Post surgery complications |  |  |  |
| Shoes didn’t fit |  |  |  |
| Shoes looked awful |  |  |  |
| Shoes too heavy |  |  |  |
| Waited a long time to be seen |  |  |  |
